# Futile attempts to differentiate provide molecular evidence for individual differences within a population of cells during cellular reprogramming

**DOI:** 10.1111/j.1574-6968.2012.02506.x

**Published:** 2012-02-15

**Authors:** Xenia-Katharina Hoffmann, Jens Tesmer, Manfred Souquet, Wolfgang Marwan

**Affiliations:** 1 Magdeburg Centre for Systems Biology (MaCS), Otto von Guericke UniversityMagdeburg, Germany; 2 Beckman Coulter GmbHKrefeld, Germany

**Keywords:** *Physarum polycephalum*, sporulation, cellular reprogramming, photomorphogenesis, cell differentiation, single-cell multiplex RT-PCR

## Abstract

The heterogeneity of cell populations and the influence of stochastic noise might be important issues for the molecular analysis of cellular reprogramming at the system level. Here, we show that in *Physarum polycephalum*, the expression patterns of marker genes correlate with the fate decision of individual multinucleate plasmodial cells that had been exposed to a differentiation-inducing photostimulus. For several hours after stimulation, the expression kinetics of PI-3-kinase, piwi, and pumilio orthologs and other marker genes were qualitatively similar in all stimulated cells but quantitatively different in those cells that subsequently maintained their proliferative potential and failed to differentiate accordingly. The results suggest that the population of nuclei in an individual plasmodium behaves synchronously in terms of gene regulation to an extent that the plasmodium provides a source for macroscopic amounts of homogeneous single-cell material for analysing the dynamic processes of cellular reprogramming. Based on the experimental findings, we predict that circuits with switch-like behaviour that control the cell fate decision of a multinucleate plasmodium operate through continuous changes in the concentration of cellular regulators because the nuclear population suspended in a large cytoplasmic volume damps stochastic noise.

## Introduction

*Physarum polycephalum*, a member of the amoebozoa group of organisms (Glöckner *et al*., 2008), has a complex meiotic life cycle involving several different cell types that occur in temporal order in response to environmental conditions (Sauer, 1982; Dee, 1987; Burland *et al*., 1993). Through mating or apogamic development, mononucleated amoebal cells can differentiate into a multinucleate single cell, a so-called plasmodium. One individual plasmodial cell as used in this study spreads over the surface of an agar plate in a 9-cm Ø Petri dish and contains between 10^7^ and 10^8^ nuclei suspended in a vigorously streaming cytoplasm. Depending on the environmental conditions, the plasmodium may stay in a proliferative state for an unlimited period of time or become committed to alternative differentiation pathways, leading to sporulation or spherulation (Schreckenbach & Verfuerth, 1982; Putzer *et al*., 1983, 1984; Martel *et al*., 1988). Sporulation can be experimentally triggered by a far-red light pulse sensed by a phytochrome-like photoreceptor (Starostzik & Marwan, 1995a; Lamparter & Marwan, 2001). About 10–12 h after the differentiation stimulus, the entire protoplasmic mass of the plasmodium synchronously develops into fruiting bodies. The nuclei in the fruiting bodies undergo a meiotic recombination for the formation of drought-resistant spores as precursors of amoebal gametes. Here, we compare the expression patterns of a set of differentially regulated genes between cells that sporulated and those that had been exposed to the same differentiation stimulus but, for unknown reasons, failed to sporulate.

## Materials and methods

### Growth and preparation of sporulation-competent plasmodia

Plasmodia of the apogamic strain WT31 (Starostzik & Marwan, 1998), which is wild type with respect to the photocontrol of sporulation, were grown for 4 days at 24 °C in 3 L of growth medium (Daniel & Baldwin, 1964) in a 5-L fermentor (Minifors, Infors HT, Bottmingen, Switzerland) inoculated with 2 % of a 3.5-day-old shaken culture, supplied with 1 L of air per minute and stirred at 250 r.p.m. with a marine propeller. Microplasmodia were harvested and applied to starvation agar plates (9 cm diameter) as described (Starostzik & Marwan, 1998). Plates were incubated for 6 days at 22 °C in complete darkness. During this time period, one multinucleate macroplasmodium develops on each plate and, by starvation, becomes competent for sporulation. The incubation temperature is critical in order to avoid unwanted spontaneous sporulation. Sporulation of competent plasmodia was induced by a far-red light pulse (30 min, ≥ 700 nm, 13 W m^−2^), generated by Concentra Weißlicht lamps (Osram, Munich, Germany) and passed through an Orange 478 combined to a Blue 627 plexiglass filter (Röhm, Darmstadt, Germany). After irradiation, plasmodia were returned to the dark and incubated for different periods of time at 22 °C. At different time points, three quarters of each plasmodium were harvested with a small glass spoon (Roth, Karlsruhe, FRG), shock-frozen in liquid nitrogen and stored at −80 °C for RNA isolation. One quarter was maintained in the dark for another 18 h to reveal the developmental decision (i.e. whether or not sporulation occurred). Control plasmodia were treated identically except that the light pulse was omitted (dark control). All manipulations were carried out under sterile conditions and under green safe light as described (Kroneder *et al*., 1999).

### Preparation of total RNA

For the extraction of total RNA, approximately 100 mg fresh weight material of a single plasmodium was used. Samples were homogenized quickly at room temperature for 10 s in 3 mL peqGOLD TriFast (PeqLab; Nr 30-2020) using a glass potter and incubated for further homogenization for 1 min at 50 °C. RNA was extracted with phenol/CHCl_3_ using 15-mL Phase Trap tubes according to manufacturer's instructions (PeqLab; Nr 30-0150A-01). Afterwards, the RNA was precipitated with ethanol in the presence of 0.3 M sodium acetate (Gurr & McPherson, 1991). Total RNA was washed in 70% ethanol and redissolved in a final volume of 180 μL of RNase- and DNase-free water (T143.2; Roth, Karlsruhe, Germany). To remove DNA contamination, the total RNA was digested with rDNase (Machery-Nagel; Nr. 740963) according to manufacturer's instructions. RNA was purified using the RNA Clean-up kit (Machery-Nagel; Nr. 740948.50) and eluted in a total volume of 240 μL RNase- and DNase-free water.

### Multiplex gene expression profiling (GeXP)

Multiplex RT-PCR was performed to simultaneously amplify 35 transcripts, and the amplification products were quantified through separation on a Beckman Coulter 8-capillary sequencer (CEQ 8800). The GenomeLab GeXP Start Kit (Beckman Coulter; A85017) along with the ThermoStart Taq Polymerase kit (Beckman Coulter; A25395) was used according to the GeXP Chemistry Protocol (Beckman Coulter, February 2009). cDNA synthesis and polymerase chain reaction were performed in half of the recommended volume leaving the recommended concentrations unchanged. Ten nanograms of total RNA was used for each reverse transcription (RT).

#### Primer design

A set of 35 genes (Table 1) was chosen from transcriptome data (Glöckner *et al*., 2008; Barrantes *et al*., 2010) as described in the Results and discussion section. Primers for the multiplexed panel were designed to generate amplified DNA fragments with similar GC contents and melting temperature and of appropriate different lengths ranging between 114 and 357 bp (Supporting Information, Table S1). Each primer contained a universal priming sequence at the 5′-end and a gene-specific sequence at the 3′-end. The concentration of each gene-specific reverse primer in the premixed reverse primer plex was experimentally adjusted to give a fluorescence signal for each fragment that was in the linear sensitivity range of the measurement system. The attenuated concentration of each reverse primer depended on its sequence and was adjusted to a value between 0.5–0.00025 μM (Table S1).

**Table 1:** Genes analysed for transcript abundance and their degree of similarity to orthologs in the Swissprot data base

Gene	Similarity	Swissprot entry	E-value	% Query coverage
*anxA*	Annexin-B12	P26256	6.00E−041	98.00
*ardA*	Actin, plasmodial isoform	P02576	9.00E−109	69.00
*arpA*	Probable basic-leucine zipper transcription factor G	Q54RZ9	2.00E−012	29.00
*cdcA*	Cell division control protein 31	P06704	6.00E−027	38.00
*cudA*	Putative transcriptional regulator cudA	O00841	1.00E−023	38.00
*damA*	DNA damage-binding protein 1a	Q9M0V3	8.00E−100	87.00
*dspA*	Dual specificity protein phosphatase 12	Q9JIM4	1.00E−012	41.00
*ehdA*	EH domain-containing protein 1	Q641Z6	6.00E−025	94.00
*gapA*	Probable GTPase-activating protein 8	Q8H100	3.00E−023	52.00
*hcpA*	Histone chaperone ASF1A	Q2KIG1	5.00E−062	82.00
*hstA*	Probable histone H2B 4	Q27876	1.00E−041	43.00
*ligA*	Checkpoint protein hus1 homologue 1 (LigA)	Q54NC0	1.00E−028	94.00
*meiB*	Meiosis protein mei2	Q64M78.1	9.00E−064	27.00
*nhpA*	Nonhistone chromosomal protein 6	Q4PBZ9	5.00E−017	30.00
*pakA*	Serine/threonine protein kinase pakC	Q55GV3	3.00E−048	79.00
*pcnA*	Proliferating cell nuclear antigen	Q43124	9.00E−076	81.00
*pikB*	Phosphatidylinositol 3-kinase 2	P54674	3.00E−063	68.00
*pikC*	Phosphatidylinositol 4-kinase beta	Q49GP3	8.00E−050	94.00
*pksA*	Serine/threonine protein kinase phg2	Q54QQ1	9.00E−035	89.00
*pldA*	Phosphatidylinositol-glycan-specific phospholipase D	Q8R2H5	4.00E−062	91.00
*pldB*	Phosphatidylinositol-glycan-specific phospholipase D	P80108	1.00E−080	83.00
*pldC*	Phospholipase D	Q9LRZ5	4.00E−014	61.00
*pptA*	Phosphatase DCR2	Q05924	6.00E−019	59.00
*pptB*	Protein phosphatase 2C POL	Q8RWN7	0.016	14.00
*psgA*	*Physarum* specific gene			
*pumA*	Pumilio homologue 2	Q80U58	2.00E−046	80.00
*pwiA*	Piwi-like protein 1	Q96J94	2.00E−055	92.00
*ralA*	Circularly permutated Ras protein 1	Q75J93.1	4.00E−017	58.00
*rasA*	Ras-related protein RABD2a	P28188	3.00E−029	49.00
*rgsA*	Regulator of G-protein signalling 2	O08849	3.00E−005	31.00
*ribA*	60S ribosomal protein L38	Q1HRT4	2.00E−017	40.00
*ribB*	60S ribosomal protein L4-2	Q54Z69	4.00E−036	66.00
*spiA*	Protein spire	Q9U1K1	1.00E−004	31.00
*tspA*	Tumour suppressor p53-binding protein 1	P70399	2.00E−004	16.00
*uchA*	Programmed cell death protein 2	Q2YDC9	1.00E−006	32.00

The genes are named following the valid rules of genetic nomenclature for *Physarum polycephalum*.

#### cDNA synthesis

The RT reaction was performed in a thermal cycler with 10 ng of total RNA, 1× RT buffer, 1 μL of reverse primer mix, 0.63 μL KAN^r^ RNA and 0.5 μL reverse transcriptase in a total volume of 10 μL with the following temperature programme: 1 min at 48 °C, 5 min at 37 °C, 60 min at 42 °C, 5 min at 95 °C, hold at 4 °C. The plate was briefly centrifuged to recollect the sample volume at the bottom of the wells.

#### Polymerase chain reaction

About 4.7 μL of the 10 μL RT reaction was used as template for the PCR. The PCR was performed with 0.35 μL Thermo-Start DNA Polymerase, 5 mM MgCl_2_, 1× PCR buffer and premixed forward primer plex (20 nM final concentration) in a volume of 10 μL under the conditions: 10 min at 95 °C, followed by 35 cycles of 30 s at 94 °C, 30 s at 55 °C and 1 min at 68 °C, hold at 4 °C.

#### Fragment analysis

Amplification products were separated and quantified according to the manufacturer's instructions. Briefly, the PCR products were prediluted 20-fold with 10 mM Tris-HCl pH 8.0. One microlitre of the diluted sample was separated with 38.5 μL sample loading solution and 0.5 μL DNA size standard-400 (Beckman Coulter) on the GenomeLab Separation Capillary Array of the CEQ8800 (Beckman Coulter) using the Frag-3 separation programme. The raw data were analysed using the Fragment analysis module of the CEQ8800 software (Beckman Coulter) to estimate the size of the obtained fragments. The peak area of each fragment was exported for subsequent data processing.

#### Calibration curves

A calibration curve for each transcript was established to calculate its relative concentration in the analysed samples from the corresponding normalized fluorescence signal values integrated over the peak areas of the respective amplified DNA fragments as separated on the capillary sequencer. To obtain the calibration curve, total RNA samples from dark- and light-treated plasmodia were pooled to give an appropriate analytical standard. The pool was repeatedly diluted, twofold in each step, to obtain a set of concentrations of total RNA ranging from 100 to 0.78 ng per reaction volume. Each sample was measured twice. The relative fluorescence signal *F*_*i*_ measured for each DNA fragment *i* was obtained by normalizing its peak area relative to the peak area of the KAN^r^ control RNA that served as internal standard. Mean values 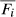 were calculated from repeated measurements. The logarithm of these normalized mean values was plotted against the logarithm of the relative total RNA concentrations as obtained in the dilution series (Rai *et al*., 2009). A straight line in the form 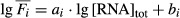 was computed by linear regression for each DNA fragment *i* where 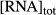 is the relative concentration of the total RNA in each sample, *a*_*i*_ is the slope of the fitted straight line, and *b*_*i*_ is its intersection with the ordinate. Linear regression was carried out in MATLAB (© 1984–2010 The MathWorks, Inc.). The slopes *a*_*i*_ of the curves for all fragments *i* were averaged, and the mean slope 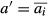 was used as a fixed value to recalculate the linear regression curves to give specific 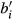 values for each fragment *i*. This procedure yielded a set of straight lines shifted parallel relative to each other on the 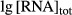 abscissa. There was no systematic deviation of the data points for each of the fragments from the mean slope *a*′. The relative transcript concentration 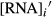 for each fragment *i* was calculated on the basis of its specific calibration curve as 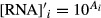 with 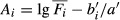 and with 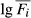 values obtained in two independent RT-PCR and independent subsequent separations.

### Gene clustering

The hierarchical clustering display dendrogram based on the Euclidean distance and the corresponding heat map were obtained using the appropriate functions of the bioinformatics toolbox for MATLAB (© 1984–2010 The MathWorks, Inc.).

## Results and discussion

### Genes predicting the commitment to sporulation through their differential regulation

The transcriptomes of sporulation-competent and sporulation-induced plasmodial cells at 6 h after an inductive far-red light pulse were established by 454 sequencing (Barrantes *et al*., 2010) aimed to identify the genes that are differentially regulated during commitment. While the 454-sequencing experiments were performed with pooled RNA samples isolated from several individual plasmodia in order to obtain a statistically representative result, we now analysed RNA samples of individual plasmodial cells to detect possible cell-to-cell variations in the gene expression patterns within an apparently homogeneous cell population (see Materials and methods). In general, the probability that an individual plasmodium will sporulate in response to a light pulse depends on the photon exposure (the number of photons applied) and varies in a dosage-dependent manner from 0% to almost 100% (Starostzik & Marwan, 1995a, b). The developmental decision of an individual plasmodium is always all or none in terms of sporulation, suggesting stochasticity in the developmental decision which, when made, evenly encompasses the entire multinucleate cell mass. The all or-none behaviour holds independent of the strength of the photostimulus. Even at saturating photon exposure, certain plasmodia may fail to sporulate. The dose–response curve typically saturates at values between 80–100% of sporulating plasmodia when grown and maintained under our experimental conditions (Starostzik & Marwan, 1995a, b; Marwan & Starostzik, 2002).

**Fig. 1:**
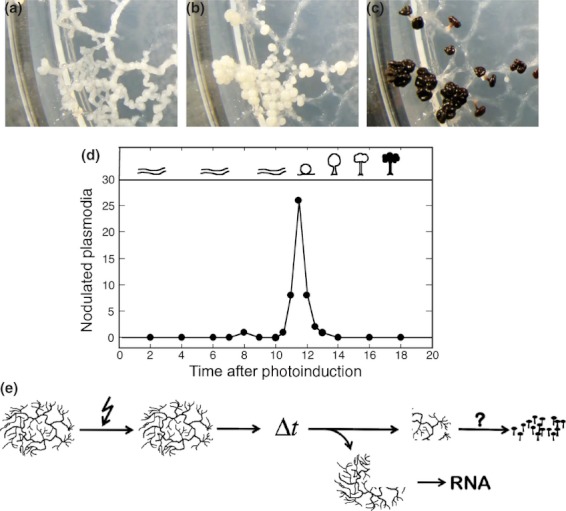
Light-induced sporulation of *Physarum polycephalum*: developmental stages, time course and sample preparation. (a-c) Morphogenetic changes leading to the development of fruiting bodies with spores. After induction of sporulation with a far-red light pulse, there is a premorphogenetic phase without any visible changes in the plasmodial morphology. Beyond 8–9 h after the light pulse, plasmodial strands wind up (a) and break apart into nodular structures (nodulation stage) (b). Each nodule culminates and differentiates into an individual melanized fruiting body (c). Inside the fruiting bodies, the protoplasm is cleaved and mononucleate haploid spores are formed after meiotic recombination. The micrographs shown here have been taken from the Movie S1. (d) Entry into the nodulation stage of individual plasmodia in a population grown and starved under the experimental conditions described in Materials and methods. The number of plasmodia that entered the nodulation stage is plotted as a function of time elapsed after exposure to a light stimulus pulse. The kinetic is typical in terms of synchrony of development (i.e. broadness of the distribution), although the onset of nodulation and the maximum of the curve may shift by approximately 1 or 2 h from experiment to experiment. (e) Experimental protocol of sample preparation. After application of a far-red light stimulus pulse of 30 min, the plasmodia are retuned to the dark. At time Δ*t* after the stimulus pulse, each plasmodium together with its supporting agar slice is cut into two pieces. Three quarters are frozen in liquid nitrogen for the subsequent isolation of RNA, and one quarter is maintained in the dark and checked for sporulation on the following day. In corresponding dark controls, the light stimulus was omitted.

Plasmodia were exposed to a 30-min far-red light pulse and returned to the dark. At different time intervals after the inductive pulse, three quarters of each individual plasmodium were frozen in liquid nitrogen for subsequent isolation of RNA and the remaining quarter was left on its supporting agar slice and returned to the dark to see the developmental decision on the next day, that is, whether the plasmodium had sporulated or not (Materials and methods) behave homogenously as judged by the synchrony with which the nodulation stage as the first visible morphogenetic response typically occurs (Fig. 1b and Movie S1). Plasmodia from the dark control were not irradiated but otherwise treated identically (and frozen at different time points).

Transcriptome data were used to select a set of genes for GeXP quantitative RT-PCR. Selection criteria were the differential regulation in response to the differentiation-inducing stimulus and the sequence similarity to genes with known function. From the list of genes that appeared to be clearly up- or downregulated in the 454-sequencing experiments, such genes were picked that are predicted to encode members of different classes of proteins regarding their role in cellular signalling or development (Table 1). In addition, genes with apparently constant expression level (*pksA*, *dspA*, *ribB*, *damA*, and *ribA*) were chosen as reference for the GeXP analysis. For each RNA sample analysed, this set of 35 transcripts together with the kanamycin standard RNA was assayed in a single multiplex RT-PCR. Each RNA sample was analysed twice. The labelled PCR products of each reaction were quantified by separation on an 8-capillary sequencer. In 664 independent experiments performed as duplicates (corresponding to 1328 samples), there was an average deviation from the means of ±9.7%, indicating the robustness of the method.

**Fig. 2:**
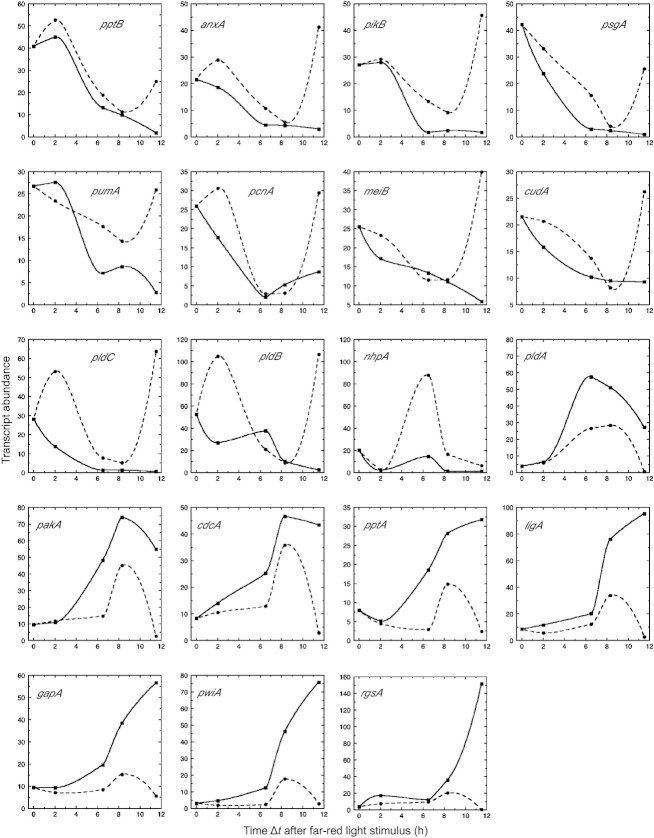
Gene expression kinetics of plasmodial cells that sporulated or did not sporulate despite receiving the same stimulus as compared to the unstimulated controls. Plasmodia were exposed to a 30-min pulse of far-red light and returned to the dark. Samples were taken at Δ*t* after the onset of the stimulus pulse as described. The relative abundance of each transcript of the different transcripts was determined by GeXP analysis and calculated as described in Materials and methods. Data were averaged and plotted separately for plasmodia that sporulated (▪; solid line) and such that did not sporulate (•; dashed line) despite they received the same stimulus. The level of the transcripts found in the unstimulated plasmodia (dark controls) is plotted as a data point at time point 0 h. The data points are connected by interpolated lines to guide the eye. Note that the data points for each time Δ*t* were obtained by averaging the values of different plasmodia and hence do not necessarily reflect the time course that would be observed by repeated sampling of an individual plasmodial cell.

Samples were taken at about 2, 6, 8 and 11 h after stimulation with the far-red light pulse, and the gene expression pattern in each individual plasmodium was analysed. Gene expression data were correlated with the occurrence or absence of sporulation. Plasmodia in which the remaining quarter finally sporulated and those in which the quarter failed to sporulate will be referred to as sporulating and nonsporulating, respectively, keeping in mind that the samples for RNA isolation were taken well before the developmental decision had become visible. The mean expression values for sporulating and nonsporulating plasmodia were plotted separately as a function of time and displayed for those transcripts where relatively large changes in expression level occurred (Fig. 2).

In sporulating plasmodia, transcripts of *pikB* (phosphatidylinositol 3-kinase), *pcnA* (proliferating cell nuclear antigen), and *pldC* (phospholipase D) among others strongly decreased, while *pldA* (glycoprotein phospholipase D), *pakA* (serine/threonine protein kinase) and *pwiA* (Piwi-like protein) and other transcripts strongly accumulated already at 6 h after light stimulation as compared to the dark controls (Fig. 2). For several transcript levels, a significant change had already occurred at 2 h (e.g. *psgA*, *pcnA, pldB*), while the largest differences between the light-stimulated plasmodia and the unstimulated (dark) controls were observed at the 11.5-h time point, shortly before nodulation started. Discrimination between immediate early and second wave genes will be addressed in a future experimental approach.

Even light-stimulated plasmodia that did not sporulate showed on average a clear response in transcript abundance for both the up- and the downregulated genes. For most genes, the change in transcript levels occurred in the same direction in sporulating and nonsporulating plasmodia. However, beyond the 8-h time point, the expression levels diverged between the two groups. The transcript levels in the nonsporulated plasmodia not only approached the values of the dark control plasmodia but in some cases even went beyond as clearly visible for *pikB*, *meiB*, *cdcA* and *pptA* (Fig. 2). For most of the transcripts showing an intensive change in abundance, the difference at the 11.5-h time point indeed was drastically between sporulated plasmodia and the two plasmodia that did not sporulate.

**Fig. 3:**
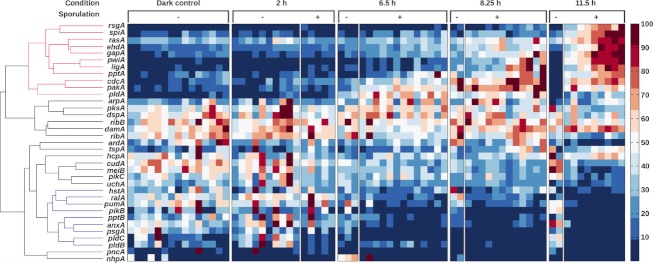
Heat map displaying the relative transcript levels of 35 genes for each individual plasmodial cell that were averaged in Fig. 2. Genes were grouped according to the similarity of their expression patterns as described in Materials and methods. Each column represents the values of one individual plasmodial cell. Columns were grouped separately for plasmodia that sporulated (+) and those that did not sporulate (−). The values for Δ*t* are given at the top of the figure. The numerical values of the transcript levels are listed in Table S2.

A heat map (Fig. 3) shows the expression levels of the 35 genes for all individual plasmodial cells as analysed and averaged in Fig. 2. The dendrogram was calculated using the values of the dark controls and of the sporulating plasmodia to identify the similarities in the expression patterns of the considered genes. It suggests five genes *pksA*, *dspA*, *ribB*, *damA* and *ribA* that only slightly if at all responded to the light stimulus and their transcripts may serve as a reference accordingly. The heat map pinpoints the drastic differences in gene expression between individual sporulating and nonsporulating plasmodial cells at the 11.5-h time point, as well as the relatively small differences between the two groups at 2 h after delivery of the light stimulus.

Although all plasmodial cells received the same light stimulation, the number of nonsporulating plasmodia was markedly higher in quarters (sporulation controls) cut-off at 2 h as compared to those cut at later time points (Fig. 3). The reduced ability of the plasmodial quarters to subsequently sporulate might be either due to the cutting itself or, more likely, to the well-known size effect, the phenomenon that otherwise identically treated large plasmodia do sporulate with a higher probability than smaller ones, eventually because of a co-operative effect between the plasmodial nuclei. It is noteworthy that even small pieces cut from committed plasmodia at six or more hours after the light stimulus usually do sporulate (our unpublished observations).

### The multinucleate plasmodium behaves like one single cell

The natural developmental synchrony of the nuclei in a multinucleate plasmodial cell has been convincingly established by thorough studies of the cell cycle (Rusch *et al*., 1966; Sachsenmaier *et al*., 1972) including experiments in which plasmodia in different phases of the cell cycle have been fused and in which cytoplasmic mixing synchronized two nuclear populations that had previously been in different phases (Rusch *et al*., 1966). In addition to cell cycle synchrony, developmental synchrony is obvious by the fact that small pieces cut from a committed plasmodium subsequently do sporulate in parallel (see above), suggesting that the decisive protein factors were evenly distributed in the plasmodium. The permanent and vigorous cytoplasmic shuttle streaming may contribute to or even cause the natural synchrony of the nuclei by mixing signalling proteins and transcription factors to clear local concentration differences. That plasmodial nuclei are synchronous not only with respect to the cell cycle but also with respect to sporulation, in addition, is suggested by the observation that individual fruiting bodies are formed simultaneously in a plasmodium that has been committed to sporulation (see Movie S1). As mentioned above, the developmental response of a plasmodium to nonsaturating light pulses is both stochastic and all-or-none, indicating that nuclear synchrony even persists in response to stimulation at threshold where stochastic effects influence cellular commitment and developmental switching.

The developmental synchrony of a plasmodial cell is evident through the differences in transcript abundance of sporulating as compared to nonsporulating cells most clearly seen at the 11.5-h time point. The *pakA*, *cdcA* and *pptA* expression levels, for example, are high in sporulating plasmodia but very low in nonsporulating plasmodia at this time point as compared to the previous (8.5 h) one. The very low relative gene expression levels in nonsporulating plasmodia suggest that the concentrations of the respective transcripts simultaneously decreased all over the entire plasmodial cell as compared to the preceding time point. If commitment and differentiation, indeed, are controlled by molecular processes that evenly spread over and uniformly engage in the entire plasmodium, the plasmodial cell could serve as a source for macroscopic amounts of synchronous protoplasm useful to study the molecular basis of the regulation of cellular commitment and of switching between proliferative and differentiated states.

### Stochastic developmental switching in a multinucleate plasmodium

Sporulation of a far-red stimulated plasmodium can be prevented by a subsequent red light pulse given not later than 2 h after the far-red pulse or by glucose fed not later than 5–6 h after far-red, suggesting the occurrence of two irreversible steps that are presumably mediated by two subsequent switch-like events (Starostzik & Marwan, 1995a, b). The data of Fig. 2 suggest that in nonsporulating plasmodia, gene expression is switched back to or even beyond the prestimulus level at about 8 h after light stimulation. This would argue for a third (irreversible) switch (point of no return). However, direct evidence at the molecular level for switching back requires the detection of changes in transcript abundance observed in samples that were taken from one and the same plasmodium at different time points after light stimulation. Measuring transcript kinetics by repeated sampling of a single cell would avoid ambiguities in the interpretation of data obtained from different cells as combined in Fig. 2, due to the fact that a potential heterogeneity within a population of seemingly identical plasmodia cannot be excluded *a priori*. Simply increasing the sample size would not help here. Anyway, the results show that in the multinucleate plasmodial cell developmental switching although occurring stochastically at the single-cell level (Starostzik & Marwan, 1995a, b, 1998; Marwan *et al*., 2005) results in drastic changes in the concentration of transcripts and presumably of other regulatory components. Because populations of (functionally coupled) nuclei suspended in a large protoplasmic volume average out stochastic fluctuations to a certain extent, switch-like events even if they should be stochastically triggered, presumably can be experimentally detected by changes in the concentration of regulatory components. These concentration changes would have a mechanistic cause and in this respect could be distinguished from stochastic noise as it might occur in a typical mononucleate single cell because of the low number of molecules contained in the small cytoplasmic volume at physiological concentration. The elimination of noise in the *Physarum* plasmodium might indeed provide complementing mechanistic information difficult to obtain in noisy mononucleate cells, even in those cases where single-cell measurements are being taken. In the absence of noise, one might even find boundary formation in response to a shallow morphogen gradient as it occurs, for example, in the developing egg of *Drosophila* or other flies which would provide additional mechanistic insight.

To this end, we identified a set of marker genes to discriminate successful and futile attempts of individual plasmodial cells to differentiate. We conclude that the plasmodial cell provides an experimental system in which the dynamics of cellular reprogramming can be analysed by monitoring the time-dependent changes in the concentrations of its molecular components in response to perturbations. The macroscopic amount of naturally synchronous protoplasm available from a single plasmodial cell even encourages the combination of time-resolved transcriptomic and proteomic approaches to analyse the dynamic processes that mediate cellular reprogramming.

## Supporting Information

**Table S1.** Names of genes and cDNA contigs and primer sequences for the amplification of the respective cDNAs in the GeXP assay.

**Table S2.** Expression values of all genes measured in all plasmodial cells as displayed in Fig. 3.

**Data S1.** The file contains the available cDNA sequence information (Glöckner *et al*., 2008; Barrantes *et al*., 2001) of the transcripts analysed in this study.

**Movie S1.** Time-lapse movie showing the fruiting body development of *Physarum polycephalum* 900-fold faster than it occurred in real time (14.25 h).

## References

[b1] Barrantes I, Glöckner G, Meyer S, Marwan W (2010). Transcriptomic changes arising during light-induced sporulation in *Physarum polycephalum*. BMC Genomics.

[b2] Burland TG, Solnica-Krezel L, Bailey J, Cunningham DB, Dove WF (1993). Patterns of inheritance, development and the mitotic cycle in the protist *Physarum polycephalum*. Adv Microb Physiol.

[b3] Daniel JW, Baldwin HH (1964). Methods for culture of plasmodial myxomycetes. Methods Cell Physiol.

[b4] Dee J (1987). Genes and development in *Physarum*. Trends Genet.

[b5] Glöckner G, Golderer G, Werner-Felmayer G, Meyer S, Marwan W (2008). A first glimpse at the transcriptome of *Physarum polycephalum*. BMC Genomics.

[b6] Gurr SJ, McPherson MJ, McPherson MJ, Quirke P, Taylor GR (1991). PCR-directed cDNA libraries. PCR. A Practical Approach.

[b7] Kroneder R, Cashmore AR, Marwan W (1999). Phytochrome-induced expression of *lig1*, a homologue of the fission yeast cell cycle checkpoint gene *hus1* is associated with the developmental switch in *Physarum polycephalum* plasmodia. Curr Genet.

[b8] Lamparter T, Marwan W (2001). Spectroscopic detection of a phytochrome-like photoreceptor in the Myxomycete *Physarum polycephalum* and the kinetic mechanism for the photocontrol of sporulation by P_fr_. Photochem Photobiol.

[b9] Martel R, Tessier A, Pallotta D, Lemieux G (1988). Selective gene expression during sporulation of *Physarum polycephalum*. J Bacteriol.

[b10] Marwan W, Starostzik C (2002). The sequence of regulatory events in the sporulation control network of *Physarum polycephalum* analysed by time-resolved somatic complementation of mutants. Protist.

[b11] Marwan W, Sujatha A, Starostzik C (2005). Reconstructing the regulatory network controlling commitment and sporulation in *Physarum polycephalum* based on hierarchical Petri net modeling and simulation. J Theor Biol.

[b12] Putzer H, Werenskiold K, Verfuerth C, Schreckenbach T (1983). Blue light inhibits slime mold differentiation at the mRNA level. EMBO J.

[b13] Putzer H, Verfuerth C, Claviez M, Schreckenbach T (1984). Photomorphogenesis in *Physarum*: induction of tubulins and sporulation-specific proteins and of their mRNAs. P Natl Acad Sci USA.

[b14] Rai AJ, Kamath RM, Gerald W, Fleisher M (2009). Analytical validation of the GeXP analyzer and design of a workflow for cancer-biomarker discovery using multiplexed gene-expression profiling. Anal Bioanal Chem.

[b15] Rusch HP, Sachsenmaier W, Behrens K, Gruter V (1966). Synchronization of mitosis by the fusion of the plasmodia of *Physarum polycephalum*. J Cell Biol.

[b16] Sachsenmaier W, Remy U, Plattner-Schobel R (1972). Initiation of synchronous mitosis in *Physarum polycephalum*. Exp Cell Res.

[b17] Sauer HW (1982). Developmental Biology of Physarum.

[b18] Schreckenbach T, Verfuerth C (1982). Blue light influences gene expression and motility in starving microplasmodia of *Physarum polycephalum*. Eur J Cell Biol.

[b19] Starostzik C, Marwan W (1995a). A photoreceptor with characteristics of phytochrome triggers sporulation in the true slime mould *Physarum polycephalum*. FEBS Lett.

[b20] Starostzik C, Marwan W (1995b). Functional mapping of the branched signal transduction pathway that controls sporulation in *Physarum polycephalum*. Photochem Photobiol.

[b21] Starostzik C, Marwan W (1998). Kinetic analysis of a signal transduction pathway by time-resolved somatic complementation of mutants. J Exp Biol.

