# Supplementary material for: Futile attempts to differentiate provide molecular evidence for individual differences within a population of cells during cellular reprogramming
Source: FEMS Microbiol Lett. 2012 Feb 15;329(1):78–86. doi: 10.1111/j.1574-6968.2012.02506.x (PMC3505798; doi:10.1111/j.1574-6968.2012.02506.x)
Supplement: Supplementary file 3 [file fml0329-0078-SD3.docx]

**Data S1.** The file contains the available cDNA sequence information (Glöckner*, et al.*, 2008, Barrantes*, et al.*, 2010) of the transcripts analysed in this study.
