# Supplementary material for: Futile attempts to differentiate provide molecular evidence for individual differences within a population of cells during cellular reprogramming
Source: FEMS Microbiol Lett. 2012 Feb 15;329(1):78–86. doi: 10.1111/j.1574-6968.2012.02506.x (PMC3505798; doi:10.1111/j.1574-6968.2012.02506.x)
Supplement: Supplementary file 4 [file fml0329-0078-SD4.docx]

**Movie S1.** Time-lapse movie showing the fruiting body development of *Physarum* polycephalum 900-fold faster than it occurred in real time (14.25 hours).
